# Impact of Efficient Resource Management Practices on Sustainable Performance: Moderating Role of Innovative Culture-Evidence From Oil and Gas Firms

**DOI:** 10.3389/fpsyg.2022.938247

**Published:** 2022-09-01

**Authors:** Yihan Wang, Shaojie Zhang, Shilin Xu

**Affiliations:** ^1^School of Business and Management, Jilin University, Changchun, China; ^2^School of Educational Science, Tonghua Normal University, Tonghua, China

**Keywords:** resource management, innovative culture, institutional theory, sustainable performance, corporate social responsibility

## Abstract

Academics and practitioners have paid close attention to waste, energy, and resource management due to growing awareness of its effects on sustainable performance. This study aims to explore the status and challenges of efficient resource management in China, an under-researched area. Moreover, it proposes a theoretical framework to fill the academic and practical gap how efficient resource management practices can build sustainable performance. This study justifies the need to explore the need of efficient resource management practices in emerging economies like China. Empirical data derived using a cross-sectional survey of 265 employees from oil and gas firms in China were used to test the theoretical framework developed from mainstream literature. Empirical findings of this study highlight the role of efficient resource management practices such as CSR, process and equipment, human resource practices, product design, and manufacturing planning which have a positive and significant impact on sustainable performance. In addition, innovative culture plays a moderating role in enhancing firms' sustainable performance. The findings suggest that there is further scope to utilize the efficient resource management practices for encouraging innovative culture to build sustainable performance. This study creates a basis for future research of building sustainable organizational performance by integrating efficient resource management practices. This study also highlights gaps in the system and provides insights into policymakers and manufacturing sector employees on holistically building a sustainable organization.

## Introduction

Due to environmental concerns, most of the organizations around the globe are expected to take responsibility for green environment or sustainable environment. According to different environmental expert the environment management is a priority for China because increasing industrialization will promote the growth of Chinese economy, but, at the same time, it demands more efficient resource management practices to achieve sustainable performance (Nawaz et al., 2021; Dar et al., 2022). With the increasing constrains on environment and resources, now it is becoming the mainstream trend of sustainable development to promote efficient resource management practices such as corporate social responsibility, process and equipment, human resource practices, product design, and manufacturing planning to reduce industrial pollution emissions (Ali et al., 2021). In this regard, stakeholders need to put pressure on manufacturing industries to be sustainable due to warnings about social issues (Iranmanesh et al., 2019a). Firms have grown awareness of the strategic importance of sustainable performance for a competitive advantage as the stakeholders demand that they will be legally and socially responsible (Mallak et al., 2018; Zailani et al., 2019). Given the existence of various worldwide environmental pollution and global warming issues, firms are under increasing pressure to make good decisions that benefit the environment and generations and consider the environmental implications of their actions (Yingfei et al., 2021).

Resource management is considered as an outgrowth of a broader accounting agenda for most organizations to achieve sustainable performance. Past researches on resource management have taken is different ways (Wurzer and Reiner, 2018; Elrhanimi and EL Abbadi, 2020). Researchers have explored the practices and techniques to minimize waste, resources, and energy to achieve sustainable performance (Orazalin and Mahmood, 2018). Another group of researchers has examined the number of challenges related to resource management to achieve a competitive advantage (Taelman et al., 2018). On the other hand, some researchers have analyzed the influence of resource management practices on the organization's financial, social, and environmental performance (Ali et al., 2019; Abubakr et al., 2020; Bilan et al., 2020). Researchers have suggested that environmental factors such as “environmental regulations, customer pressure, social responsibility, and environmental uncertainty” play an important role in the environmental efforts of manufacturing firms (Orazalin and Mahmood, 2018). However, past literature on using resource management practices to build sustainable performance covered many aspects of the literature, and a closer analysis of previous studies discloses serval gaps (Wurzer and Reiner, 2018; Elrhanimi and EL Abbadi, 2020).

There are a number of factors that play an important role in developing efficient resource management practices such as process and equipment, manufacturing planning and control, human resource practices, product design, and corporate social responsibility on sustainable performance. In this context, the phrase sustainable performance refers to “environmental procedures and standards imposed on businesses by institutions in order to encourage them to implement environmental initiatives” (Orji, 2019). Prior research has shown that factors such as customer pressure, excepted business benefits, environmental uncertainty, and environmental regulatory pressure have significant impacts on sustainable performance, such as environmental, social, and economic performance (Iranmanesh et al., 2019b). For instance, Mojarad et al. (2018) found that reducing greenhouse gas emissions improves long-term firm performance. To address all these gaps, this study used waste, energy, and resource management as a “second-order construct” and sustainable performance with a moderating effect of innovative culture.

However, innovative culture is a time-consuming process, which is used to transform successfully toward resource management (Habidin et al., 2018; Dorval et al., 2019; Iranmanesh et al., 2019a). According to Orazalin and Mahmood (2018), innovative culture is required criteria for manufacturing firms to achieve their goals. The workers' understanding of management methods, together with the current behavior that is done, can be considered an innovative culture (O'Cass and Ngo, 2007; Arora et al., 2020; Job et al., 2020; Aray et al., 2021). Although prior research has highlighted the importance of innovative culture in implementing resource practices, to our understanding, no research has been done on the role of innovative culture in increasing the effects of resource practices on a firm's performance (Iranmanesh et al., 2019b). Innovative culture allows firms to choose different options to satisfy their customers on a sustainable basis, so this will provide a basis for survival. Moreover, there seems to be an agreement in the literature on an innovative culture that addresses the underlying feature of the culture such as why things turn out the way they do. So, this study aims to explain how waste, energy, and resource management, as well as an innovative culture, contributes to a firm's sustainable performance (Iranmanesh et al., 2019b).

According to Cremiato et al. (2018), management practices improve firms' performance by reducing the managerial price of emission reduction, either by giving knowledge on the necessity and value of emission reduction or by decreasing the price of promoting sustainability improvements. The innovative culture is the most important factor that impacts a firm's sustainable performance (O'Cass and Ngo, 2007; Škerlavaj et al., 2010; Dorval et al., 2019; Ur Rehman et al., 2019). In conclusion, the research has demonstrated a positive association between management practices and a firm's sustainable performance. The study also has managerial importance because its findings can help managers implement appropriate waste, energy, and resource management dimensions to improve their companies' long-term success. Moreover, recognizing the effects of resource management on sustainable performance can assist manufacturing directors in realizing that resource management will enhance their company's overall performance.

This paper is divided into different parts: the first part is about the introduction, the second part deals with the literature review, the third part of the study will shine the spotlight on methodology, how to collect data, etc., the fourth part deals with the analysis of data in detail and at the end, and fifth part concentrates on discussion. In the final part, the implication of the study, future recommendations, and conclusion have been presented.

This study is based on an important theory (institutional theory) that evaluated the relationships between management practices, innovative culture, and sustainable performance.

### Institutional Theory

Institutional theory is “an approach to understand organizations and management practices as the product of social rather than economic pressures. It has created a popular perspective within management theory because of its ability to explain organizational behaviors that defy economic rationality” (Horodnic, 2018). Institutional theory can be explained into different perspectives (Simoni et al., 2020). First, coercive management refers “to both formal and informal political and regulatory influence from government or other powerful organizations or the society” (Mnif Sellami et al., 2019). “Martínez-Ferrero and García-Sánchez (2017)” stated that organizations are expected to adopt a different managerial practice due to coercive pressures. If a corporation fails to respond to this demand, its identity may be compromised. Second, normative management refers to “recognized standards that dictate what behavior is acceptable in various activity sectors, such as the values and norms held by professional associations” (Han et al., 2019). Finally, mimetic management refers “to a specific behavior in an organization's market and the perceived success of competing organizations that have already adopted it” (Han et al., 2018).

In the light of institutional theory, this study evaluates management practices such as corporate social responsibility, process and equipment, human resource practices, product design, and manufacturing planning of oil and gas manufacturing firms. Process and equipment are the characteristics to enhance the reliability, validity, time reduction, and other practices with a perspective of management. A previous study is used to process and equipment with lean practices and improve firms performance (Giantari et al., 2022). Human resource practices are included in normative management because it dedicates the organization's behavior, norms, and values of customers and employees. Through training and development, human resource practices enhance the performance of sustainable organization (Miller et al., 2019). Finally, according to the research, corporate social responsibility is a type of mimetic management that significantly impacts enterprises' decisions to adopt environmental practices (Habidin et al., 2013).

### Waste, Energy, and Resource Management

Waste, energy, and resource management is one of the methods for achieving long-term process. In today's world, humans use energy, resources, and waste at high rates that outpace the natural restructuring and assimilation capacity, resulting in environmental pollution and global warming (Avotra et al., 2021). As a result, significant improvements in efficient use of energy and waste and emission decreases are required. Manufacturing firms consume a substantial quantity of energy and resources and produce a large quantity of waste (Abubakr et al., 2020). As a result, businesses needs to adapt these methods and include waste, energy, and resource management into all aspects of the business. In this work, the resource management approach, which included “waste management, energy management, and resource management,” was represented as a second-order construct. Waste management refers to practices such as element evaluation, the 4R program “reduce, reuse, recycle, and recover.” Das and Hassan (2021) focused on reducing resource usage and waste manufacturing. Energy management includes techniques such as “energy conservation programmes, frequent energy audits, the use of electricity devices, and the use of sustainable power to reduce energy use and its environmental impact” (Aray et al., 2021). Resource management refers to methods that “try to maximize the use of resources, such as prioritizing washable, fixable, consumable, regenerative, and compostable items, conducting frequent process flow assessments, and establishing a transparent strategy” (Taelman et al., 2018).

### Innovative Culture

Innovative culture is characterized as a unique, demanding work atmosphere that is focused on results and is characterized by industrial ambition, vulnerability, and challenging behavior. It is defined as “the pattern of shared values and beliefs that help individuals understand organizational functioning and thus provide them with norms for behavior in the organization” (Iranmanesh et al., 2021). Nonetheless, experts believe that innovative culture is primarily concerned with the trends of ideas and values that are reflected in the attitudes, practices, and other items shared by a firm's employees. Past studies show strong relationship between innovative culture and innovation capabilities (Ur Rehman et al., 2019). Other prior studies show significant relationship between innovative culture and organizational leadership (Iranmanesh et al., 2021). Furthermore, administrators in participating employees' responses to specific strategic decisions reduce the chances of unexpected consequences.

Innovative culture supports other resources in achieving excellence and, as a result, improving sustainable performance (O'Cass and Ngo, 2007). A few of the key sources of change have been emphasized as the innovative culture as an element of the corporate culture. Innovative culture also described as “the shared common values, beliefs and assumptions of organizational members that could facilitate the product innovation process” (Iranmanesh et al., 2019a).

### Hypothesis Development and Framework

**Figure 1:**
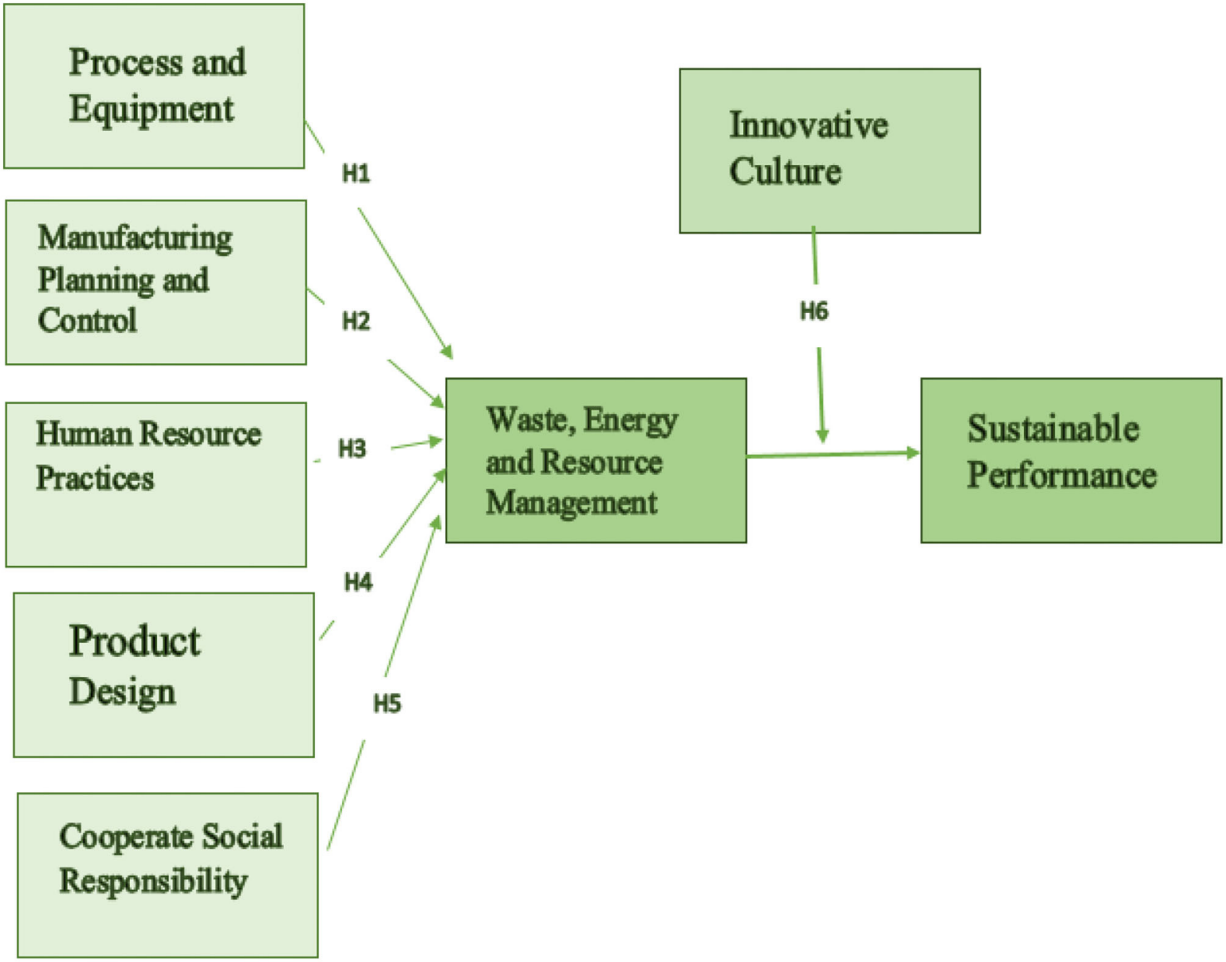
Conceptual framework.

Based on the review of the literature and according to institutional theory, this study factors include resource management practices such as corporate social responsibility, process and equipment, human resource practices, product design, and manufacturing planning and sustainable performance. In contrast, the study suggested that innovative culture had a moderating influence. Based on the literature review and theories, a conceptual framework has been developed as shown in Figure 1.

#### Process and Equipment

Processes and equipment trigger an increase in performance and procedures such as “error-proof” equipment, time savings, device reliability and security, flexible manufacturing systems, and shorter setup times to generate a normal and consistent flow within manufacturing procedures (Elrhanimi and EL Abbadi, 2020). Over-processing and incorrect processing result in material waste and defects (Iranmanesh et al., 2019b). Over-processing results in increased employee action and, as a response, mobility wastage. Processing equipment also provides “harmful compounds and pollutants, increases water use, and wastes energy (Dorval et al., 2019).” Process and equipment practices, according to Ali et al. (2019), result in fewer deficiencies and energy consumption, provide a technique for achieving sustainable and improved coordination with working atmosphere, and help to determine overflows and leakages more rapidly, reducing inorganic and resource usage and environmental damage. In conclusion, by minimizing materials and energy and also waste production, process and equipment techniques can improve environmental and firms' performance (Aray et al., 2021). Sustainable performance is expected to increase as a result of a decrease in the atmospheric consequences of firms' efforts, resulting in the benefits to the community and employees. In conclusion, by minimizing material and energy usage as well as waste disposal, process and equipment techniques can improve sustainable performance. Due to a decrease in the environmental effects of companies' activities, performance is supposed to enhance, resulting in the improvements in the community's and workers' health. As a result, it is proposed that:

***H1:*** *The process and equipment used in waste, energy and resource management effects on sustainable performance*.

#### Manufacturing Planning and Control

Manufacturing planning and control processes are often tied to scheduling approaches in management practices with the goal of coordinating productivity and demands (Iranmanesh et al., 2019b). Rapid planning and control, in relation to existing production, helps greatly to minimize the waste, raw material use, and labor usage, which are the key waste sources (Nawanir et al., 2020). Effective schedules contribute in “cost reductions, work in process reduction, resource optimization, and meeting orders” (Elrhanimi and EL Abbadi, 2020). Smaller portion also results in a better flow of production by avoiding production line imbalances. Size reduction can help decrease stocks and consequently decrease waste from overproduction (Arora et al., 2020). Planning and control procedures, according to Iranmanesh et al. (2019b), lead to a decrease in material and component utilized throughout the manufacturing process without impacting delivery schedule. The pull strategy, according to Nawanir et al. (2020), reduces work in production and floor area utilization while also eliminating wastage due to damaged products. In conclusion, by minimizing in-process waste and faulty inputs and enhancing planning and control procedures may lead to improved firms' performance. As a result, the following hypothesis has been proposed:

***H2:*** *The planning and control effects on sustainable performance*.

#### Human Resource Practices

Human resource practices are focused on supporting waste, energy, and resource management through human capital development and by giving a better environment. Employee' empowerment, attachment, autonomous issue resolving, self-directed teamwork, decision-making groups, and formal education programs are all the examples of resource practices. Human resources are continuous improvement programs, which are the key to effective waste, energy, and resource management (Nawanir et al., 2020). Human resource practice is “a series of different but interrelated activities, functions, and processes that are directed to attract, develop, maintain, and even terminate the organization's human resources” (Silvia et al., 2020). Human resource practices are the primary means through which companies influence and develop human talents, beliefs, and activities to accomplish corporate goals. Human resource practices, according to Saad et al. (2019), play a crucial role in environmental pollution reduction programs. Employees who have been trained and taught have a better awareness of waste reduction options and how to use materials effectively (Habidin et al., 2013). Other human resource practices, such as employee participation and collaboration, provide employees the ability to decrease waste and pollution by taking appropriate action, which can improve a company's performance. As a result, the following hypothesis emerges:

***H3:*** *The human resource practices effects on sustainable performance*.

#### Product Design

Product design refers to “practices such as multifunctional design teams, design for manufacturability, product modularization, and parts standardization” (Pal et al., 2019). Product design attempts to simplify the development process by reducing the quantity of material utilized in a products, which simplifies the assembling and manufacturing conditions (Dorval et al., 2019) and, as a result, maximizes the use of businesses' resources (Elrhanimi and EL Abbadi, 2020). Moreover, product design reduces energy consumption by ensuring that the design is compatible with current production methods and procedures (Habidin et al., 2018). Product design can be conceived of as a way for developing a recyclable platform that eliminates all sorts of waste during the manufacturing process (Mallak et al., 2018; Wurzer and Reiner, 2018). As a result, components of a single product can be readily separated and reassembled to create a wide range of starting point. Furthermore, to attain efficiency, which is the primary goal of lean techniques, all sorts of waste must be removed throughout the entire process (Job et al., 2020). According to Habidin et al. (2018), there is a link between product design and firm performance. As a result, the following hypothesis emerges:

***H4:*** *The product design effects on sustainable performance*.

#### Corporate Social Responsibility

Social responsibility refers to “corporations have an ethical responsibility to treat the public and the environment with dignity and respect” (Islam et al., 2021). According to Mallak et al. (2018), to comply with the beliefs and rules of society through which a corporation's behavior is dictated, the organization is likely to believe that it must make a committed dedication. As a result, Abdullah et al. (2016) claimed that social expectations were a major determinant of environmental behavior. Furthermore, Iranmanesh et al. (2019a) discovered that in developing countries, corporate social responsibility has a major impact on enterprises' decisions to undertake eco-design efforts. The idea of corporate social responsibility has gotten a lot of attention in the last decades, and its importance in the business world is growing all the time (Arora et al., 2020). Corporate social responsibility operations are influenced by a variety of issues, including the state of the economy, rules and regulations, business culture and attitude, and market competitiveness (Islam et al., 2021). Corporate social responsibility policies greatly improve an organization's ability to gain a competitive edge and achieve long-term growth goals (Ali et al., 2019). Furthermore, Iranmanesh et al. (2019a) discovered that in both developing and developed countries, social responsibility has a major impact on enterprises' decisions to undertake eco-design efforts. As a result, we anticipate that, to fulfill their social responsibility, businesses will adopt environmental practices such as “reducing energy waste and carbon dioxide emissions” (while also increasing productivity), as well as reducing resource consumption to reduce the impact on future generations. We suggest the following hypothesis based on above discussion.

***H5:*** *Corporate social responsibility effects on sustainable performance*.

#### Moderating Role of Innovative Culture

Nawaz Khan et al. (2019) have claimed that innovative culture has a significant impact on sustainable performance. Many people have characterized innovative culture (O'Cass and Ngo, 2007; Škerlavaj et al., 2010; Dorval et al., 2019; Ur Rehman et al., 2019). Moreover, there seems to be an agreement in the literature on innovative culture that addresses the underlying feature of the culture: why things turn out the way they do. Ur Rehman et al. (2019) define innovative culture as “the pattern of shared values and beliefs that help individuals to comprehend organizational functioning and so provide them with norms for behavior in the organization”. Innovative culture has been classified into four forms based on two major aspects (internal–external and organic–mechanistic): tribe, authority structure, business, and hierarchical. External placement is emphasized in the clan culture, which promotes organic processes. That is, organizations with a strong innovation appear to promote not only business, innovation, risk-taking, and staff adaptation, but also elasticity and flexibility. As a result, innovative culture increases an organization's ability to innovate, allowing it to be market-driving (Škerlavaj et al., 2010). As a result, a creative culture that creates possibilities by driving the business with innovative products has the ability to drive product performance at a higher level than those that merely attempt to respond to the industry.

To transition from industrial manufacturing administration to an innovative culture, modifications in standards and values are required. It is impossible to achieve the potential elements of business procedures without first altering the culture of the company. According to Škerlavaj et al. (2010), eliminating waste and improving the manufacturing process are not only possible through the use of energy, waste, and resource management, but also requires a company-wide culture of continuous focused on waste minimization. Furthermore, there were many businesses regarding energy, waste, and resource management as a technique rather than a concept, resulting in low implementation rates. We suggest the following hypothesis based on this:

***H6:*** *Innovative culture positively moderates the impact on process and equipment, product design, corporate social responsibility, human resource practices, and manufacturing planning and control practices on sustainable performance*.

## Methodology

Based on a thorough assessment of the literature and institutional theory, the study's theoretical framework (refer to Figure 1) has created, and hypotheses are offered.

### Participants and Procedure

This study's includes oil and gas manufacturing companies in China. The information was gathered from Chinese oil and gas executives, internal employees, and workers. They have skills and knowledge of their firms' energy, waste, and resource management, as a result of their actual participation in the production process, and hence can reply to the survey's items. Because energy, waste, and resource management is a multifaceted approach that has gradually expanded from the production industry to other company operations such as help of industrial production, supply chain, and accessing the system, the managers were chosen from various organizational departments. Questionnaires were addressed to 500 selected respondents at oil and gas companies, and 265 usable replies were received.

### Sample and Data Collection

Choosing the right sample is always regarded as a critical component for the success of any research. This study conducted in different manufacturing industries of China. Major manufacturing companies were chosen because they are environmentally conscious and are under pressure from the government, non-governmental agencies, and consumers to operate sustainably. The executives, internal employees, and workers at manufacturing companies were the targets of this research. These individuals were chosen because they are knowledgeable about waste, energy, and resource management methods and their companies' performance. The convenient sampling method is used to collect the data from the employees of manufacturing companies.

### Measurements of the Study

The items of this study adapted from previous studies. A total of 32 items were included in this study. A 5-item scale of process and equipment was adapted from the study of Iranmanesh et al. (2019b). A 3-item scale of manufacturing planning and control was adapted from the study of Iranmanesh et al. (2019b). A 5-item scale of human resource practices was adapted from the study of Iranmanesh et al. (2019b). A 4-item scale of product design was adapted from the study of Iranmanesh et al. (2019b). A 4-item scale of corporate social responsibility was adapted from the study of Islam et al. (2021). A 5-item scale of innovative culture was adapted from the study of Škerlavaj et al. (2010). A 7-item scale of sustainable performance was adapted from the study of Iranmanesh et al. (2019b). The results were collected by a “5-point Likert scale ranging from 1 = strongly disagree to 5 = strongly agree.”

## Data Analysis And Results

In Smart-PLS, there are two types of model used (measurement model and second structural model) (Hair et al., 2019). The data were analyzed using Smart-PLS 3.0 software. This software is frequently used to simulate structural equations modeling (SEM) (Hair et al., 2020). To analyze the data validity and dependability, a measurement model is used in the first stage. Factor loadings for each item, average variance extracted (AVE), heterotrait-monotrait ratio (HTMT ratio), and Fornell and Larcker criterion are used to assess the data's validity (Sarstedt et al., 2020). The Cronbach's alpha and composite reliabilities, on the other hand, are used to determine data reliability (Hair et al., 2019). The hypothesis is then tested using a structural model, in which the values of “*t*-statistics, sig-values, the sample mean, and standard deviation” are used to analyze the study (Hair et al., 2020).

### Demographic

There are total of 56.6% male respondents accounted as compared to 43.4% for females, as shown in Table 1. Men have a great influence on women in Chinese culture, particularly in organizations. When it comes to education, the majority of respondents 47.1% are pursuing bachelor's degrees, followed by master's degrees 28.4%, and others 24.5%. Furthermore, ≈56.6% of the responses were the age of 20–35 and 30% of below 20, whereas the remaining 13.3% were above 35. Moreover, ≈45.2% of the responses were having more than 5 years of experience and 35.9% having 2 years of experience, whereas the remaining 18.9% having 1 year of job experience. Finally, ≈51.0% of the responses were collected from internal employees and 26.4% from executives, whereas the remaining 22.6% collected from workers of firm.

**Table 1:** Demographic analysis.

**Demography**	**Description**	**Responses**	**%**
Gender	Male	150	56.6
	Female	115	43.4
Qualification	Bachelors	125	47.1
	Masters	75	28.4
	Ph.D. or others	65	24.5
Age	Below 20	80	30.1
	20-35	150	56.6
	Above 35	35	13.3
	1 year	50	18.9
Job Experience	2 year	95	35.9
	More than 5 years	120	45.2
Job Description	Executives	70	26.4
	Workers	60	22.6
	Internal employees	135	51.0

*N=265*.

### Measurement Model

Measurement model is defined as, “the relationship between the observed variables or indicators and the latent variables” (Sarstedt et al., 2020). According to their rule of thumb, the outer loading should be 0.5 and above and the average variance extracted should be larger than 0.5 (Hair et al., 2020). According to the following argument, starting with the lowest value, all items in the outer loading that are <0.5 should be deleted one by one.

#### Factor Loadings, Reliabilities, and AVE

**Figure 2:**
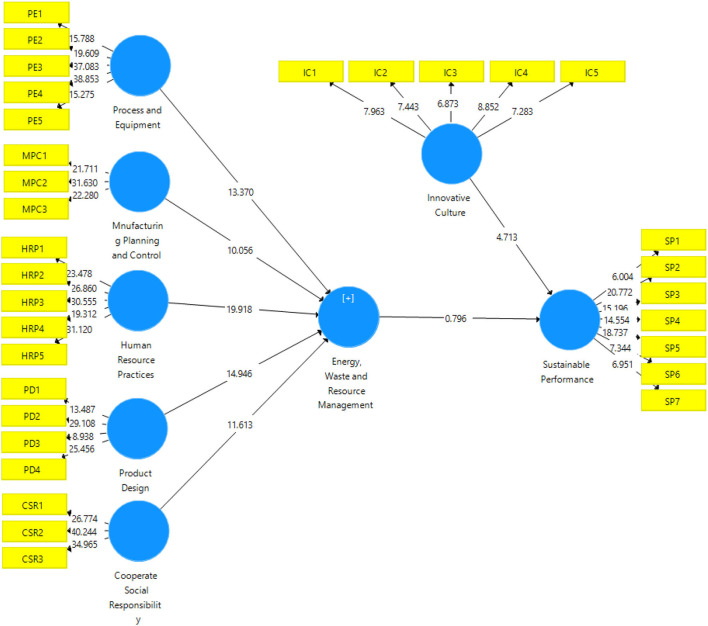
Output of measurement model algorithm.

Furthermore, the measurement model (refer to Figure 2) was used to calculate “Cronbach's Alpha (CA) and composite reliability (CR)” to examine the coherence of the measurements. “CA and CR values more than 0.7” were found in all investigation items, indicating that they met the reliability criterion (Hair et al., 2020). Factor loading levels have been classified into three categories by Lamber et al. (1991), namely, “unattractive (value 0.3), acceptable (value > 0.5), and extremely desired (value > 0.7)” (Sarstedt et al., 2020). For the current investigation, the factor loading standard was 0.5 and above. As a result, each item contributed a great deal to this study.

In each variable, Cronbach's alpha value is above the criterion (varying from 0.823 to 0.954), suggesting that the variables were extremely trustworthy. Composite reliability has a range of 0–1 and is separated into three categories: “acceptable reliability (0.6),” “satisfactory reliability (0.6–0.7),” and “highly satisfactory reliability (0.6–0.7)” (Sarstedt et al., 2020). Table 2 shows that the composite reliability value for each construct is >0.6, indicating that the composite reliability is excellent. Additionally, AVE looks for convergent validity, and its value must be >0.5 (Lamber et al., 1991). The value of AVE for each construct is more than 0.5, showing the presence of convergent validity, as shown in the Table 3.

**Table 2:** Factor loadings, reliabilities, and AVE.

	**Cronbach's Alpha**	**rho_A**	**Composite Reliability**	**(AVE)**
Corporate Social Responsibility	0.773	0.776	0.869	0.688
Energy, Waste and Resource Management	0.904	0.911	0.917	0.698
Human Resource Practices	0.868	0.869	0.905	0.655
Innovative Culture	0.633	0.622	0.766	0.595
Manufacturing Planning and Control	0.707	0.709	0.837	0.631
Process and Equipment	0.826	0.832	0.878	0.592
Product Design	0.672	0.689	0.805	0.514
Sustainable Performance	0.780	0.795	0.841	0.534

**Table 3:** Discriminant validity.

	**CSR**	**EWRM**	**HRP**	**IC**	**MPC**	**PE**	**PD**	**SP**
CSR	0.830							
EWRM	0.601	0.809						
HRP	0.545	0.804	0.849					
IC	0.359	0.569	0.565	0.798				
MPC	0.319	0.642	0.503	0.448	0.794			
PE	0.293	0.765	0.595	0.383	0.397	0.770		
PD	0.612	0.743	0.548	0.359	0.393	0.401	0.717	
SP	0.188	0.286	0.182	0.417	0.216	0.288	0.223	0.659

*CSR, Corporate social responsibility; EWRM, energy, waste, and resource management; HRP, human resource practices; IC, innovative culture; MPC, manufacturing planning and control; PE, process and equipment; PD, product design; SP, sustainable performance*.

#### Discriminant Validity

According to Fornell and Larcker (1981), discriminant validity refers to “the amount to which a given latent variable differs from other latent variables.” It was calculated by looking at the correlation between the latent variables item and the actual number of AVEs, when establishing discriminant validity recommends using latent components with a value of 0.50 or higher (Sarstedt et al., 2020).

Discriminant validity explains whether or not one construct is distinct from another. There is a discriminant validity relationship between two variables. Likewise, “the square root of every variable's (AVE) must be greater than the highest relationship of the construct with the other latent variable to assess the discriminant validity of the construct using the Fornell and Larker Criterion” (Sarstedt et al., 2020). The Table 3 demonstrates that the value at the head of each column is greater than the amount below it, showing that the variables have discriminant validity.

### Structural Model

**Figure 3:**
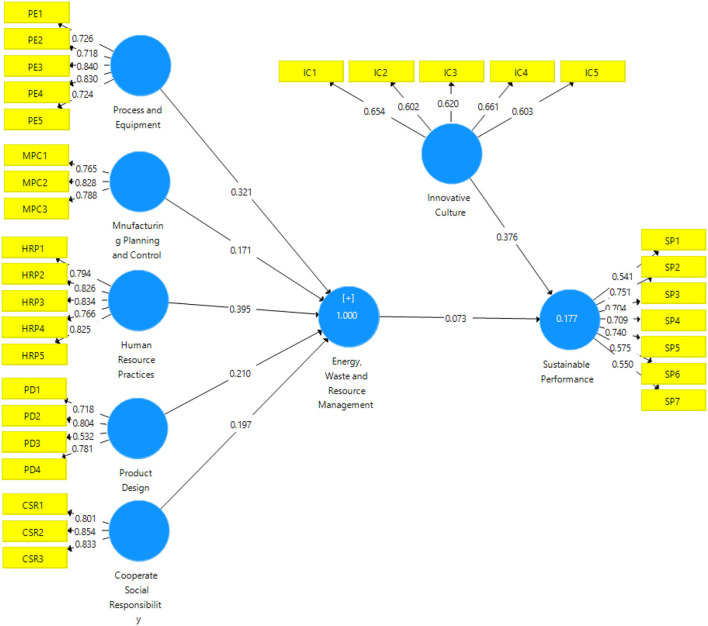
Output of structural model bootstrapping.

The structural model (refer to Figure 3) is assessed when the measurement model's criteria are completed, as mentioned above. This study's data analysis necessitates a thorough review of the structural model. PLS-SEM bootstrapping is used to examine the direct association between the study variables. The model includes coefficient and *p*-value values. For this investigation, a 95% discrimination bootstrap was used to examine the direct association between variables (Lamber et al., 1991). Structural equation modeling (SEM) is a multivariate statistical technique that allows researchers to estimate and test causal relationships (Nawaz et al., 2019).

#### Direct Hypothesis Testing

The proposed model for the study uses a structural model to stress the interconnectedness of the relationships. The structural model in PLS looks at the direct relationship between the offered hypotheses, their *t*-values, and regression coefficients. An indirect effect is the same as a standardized beta value in regression analysis (Hair et al., 2019). The *t*-values and beta values of the regression coefficients are used to determine significance; *t*-values more than “1.64” are statistically significant and are then used to make conclusions about the suggested hypothesis (Sarstedt et al., 2020). The model's two main purposes are to examine direct linkages and to verify projected interactions between components using a structural model. In Table 4, all hypothesis were accepted because *p*-value is < 0.25.

**Table 4:** Direct hypothesis testing.

	**Original Sample**	**(M)**	**STDEV**	***T* Value**	***p*-Values**
CSR → EWRM	0.197	0.196	0.017	11.613	0.000
EWRM → SP	0.173	0.176	0.091	4.796	0.005
HPR → EWRM	0.395	0.396	0.020	19.918	0.000
IC → SP	0.376	0.386	0.080	4.713	0.000
MPC → EWRM	0.171	0.168	0.017	10.056	0.000
PE → EWRM	0.321	0.321	0.024	13.370	0.000
PD → EWRM	0.210	0.209	0.014	14.946	0.000

*CSR, Corporate social responsibility; EWRM, energy, waste, and resource management; HRP, human resource practices; IC, innovative culture; MPC, manufacturing planning and control; PE, process and equipment; PD, product design; SP, sustainable performance*.

In above table, the study's 1st hypothesis is corporate social responsibility, which directly impacts on energy, waste, and resource management, so the “*p*-value = 0.000,” it shows that the hypothesis was accepted. The 2nd hypothesis is energy, waste, and resource management, which directly impacts on sustainable performance, so the “*p*-value = 0.005,” it shows that the hypothesis was accepted. The 3rd hypothesis is human resource practices, which directly impacts on energy, waste, and resource management, so the “*p*-value = 0.000,” it shows that the hypothesis was accepted. The 4th hypothesis is innovation culture, which directly impacts on sustainable performance, so the “*p*-value = 0.000,” it shows that the hypothesis was accepted. The 5th hypothesis is manufacturing planning and control, which directly impacts on energy, waste, and resource management, so the “*p*-value = 0.000,” it shows that the hypothesis was accepted. The 6th hypothesis is process and equipment, which direct impacts on energy, waste, and resource management, so the “*p*-value = 0.000,” it shows that the hypothesis was accepted. The 7th hypothesis is product design, which directly impacts on energy, waste, and resource management, so the “*p*-value = 0.000,” it shows that the hypothesis was accepted.

#### Mediating Hypothesis Testing

An analysis of mediation was “used to link between the independent and dependent variables” (Sarstedt et al., 2020). In Table 5, all hypotheses were accepted because *p*-value is < 0.25.

**Table 5:** Mediating hypothesis testing.

	**Original Sample**	**(M)**	**STDEV**	***T* Value**	***p-*Values**
HRP → EWRM → SP	0.376	0.386	0.080	5.797	0.000
PD → EWRM → SP	0.197	0.196	0.017	4.797	0.000
CSR → EWRM → SP	0.171	0.168	0.017	8.795	0.000
MPC → EWRM → SP	0.376	0.386	0.080	6.816	0.000
PE → EWRM → SP	0.395	0.396	0.020	3.770	0.000

*CSR, Corporate social responsibility; EWRM, energy, waste, and resource management; HRP, human resource practices; IC, innovative culture; MPC, manufacturing planning and control; PE, process and equipment; PD, product design; SP, sustainable performance*.

In Table 5, the first hypothesis based on energy, waste, and resource management mediates the relationship between “human resource practices and sustainable performance”, so the “*p*-value = 0.000,” it shows that the hypothesis was accepted. The study's second mediation hypothesis based on energy, waste, and resource management mediates the relationship between product design and sustainable performance, so the “*p*-value = 0.000,” it shows that the hypothesis was accepted. The study's third mediation hypothesis based on energy, waste, and resource management mediates the relationship between corporate social responsibility and sustainable performance, so the “*p*-value = 0.000,” it shows that the hypothesis was accepted. The study's fourth mediation hypothesis based on energy, waste, and resource management mediates the relationship between manufacturing planning and control and sustainable performance, so the “*p*-value = 0.000,” it shows that the hypothesis was accepted. The study's last mediation hypothesis based on energy, waste, and resource management mediates the relationship between process and equipment, and sustainable performance, so the “*p*-value = 0.000,” it shows that the hypothesis was accepted.

#### Moderating Hypothesis Testing

An analysis of moderation was “used to discover which moderator variable changes the success or strength of the link between the independent and dependent variables” (Sarstedt et al., 2020). In Table 6, all hypothesis were accepted because *p*-value is < 0.25.

**Table 6:** Moderating hypothesis testing.

	**Original Sample**	**(M)**	**STDEV**	***T* Value**	***p*-Values**
**EWRM** *IC ** → SP**	−0.084	0.043	3.279	0.007	Accept

*EWRM, Energy, waste, and resource management; IC, innovative culture; SP, sustainable performance*.

In Table 6, the study's moderating hypothesis innovative culture moderates the relationship between energy, waste, and resource management and sustainable performance, so the “*p*-value = 0.000,” it shows that the hypothesis was accepted.

#### Assessment of R^2^

The value of *R*^2^ ranges from zero to one. Moreover, Sarstedt et al. (2020) recommended that the *R*^2^ of “0.13 is considered weak,” “0.33 is moderate,” and “0.67 is considered as strong.” R Square “explains the variance in the endogenous variable explained by the exogenous variable.” In below table, energy, waste, and resource management value of R Square (1.000) considers as strong and sustainable performance value of R Square (0.177) considers as weak.

## Discussion

This study analyzed the impact of efficient resource management on sustainable performance and moderating role of innovative culture. It produced some intriguing outcomes. Similar studies were carried out to assess the organizational approaches used in sports to get beneficial results. Moreover, according to demographic data, males are more likely than females to work for oil and gas companies in China. In previous studies, other factors use energy, waste, and resource management and some indirect effects through lean culture with sustainable performance (Habidin et al., 2018; Das and Hassan, 2021). In this study, researcher investigates the direct and indirect effects with sustainable performance and moderating effect of innovative culture on sustainable performance.

This research was based on a number of theories about resource management links, such as innovative culture and sustainable performance. The 1st hypothesis evaluated the effect of component of energy, waste, and resource management as process and equipment positive impact on sustainable performance. To generate a regular and consistent flow within manufacturing processes, some techniques are required, such as process improvement, shorter setup times, ordering and maintenance, and the use of “error proof” process and equipment. The 2nd hypothesis evaluated the effect of component of energy, waste, and resource management as manufacturing planning and control positive impact on sustainable performance. There is a significant impact of manufacturing planning and control which manufacture and control pollutants. In the previous study, there is no significant effect with manufacturing planning and control with sustainable performance (Habidin et al., 2018). According to Das and Hassan (2021), lot of reduction in size as a manufacturing planning and control method necessitates frequent interchange, which results in more unneeded process material being discarded and increased employee's stress. According to Mallak et al. (2018), the negative effect of manufacturing planning and control techniques such as Kanban and production management outweigh their environmental benefits.

The 3rd hypothesis evaluated the effect of component of energy, waste, and resource management as human resource practices positive impact on sustainable performance. The human resource practices train and develop employees to improve sustainable performance. Human resource practices explained why this association exists, claiming that skilled personnel have a greater understanding of resources and can provide suitable solution, resulting in the reduced material waste. This conclusion contradicts the findings of Orji (2019), who found that human resource practices reduce environment pollution output. Orji (2019) also emphasized the importance of workers in pollution prevention. Experts, administrators, and production employees may all contribute to reducing waste and improving environmental results by working together as a team. The 4th hypothesis evaluated the effect of component of energy, waste, and resource management as product design positive impact on sustainable performance. Product design practices aimed at eliminating useless processing steps and standardizing the manufacturing process can help to optimize resource usage and improve effectiveness and productivity by reducing activities and waste throughout the overall process, as well as reduce work stress. Companies use product design principles to limit waste formation, and as mentioned by Habidin et al. (2018), waste reduction is the main emphasis of approaches to reduce environmental effect.

The 5th hypothesis evaluated the effect of component of energy, waste, and resource management as corporate social responsibility positive impact on sustainable performance. The importance of corporate social responsibility in encouraging businesses to be concerned about environmental issues. The corporate social responsibility connection is one of the factors of the implementation and distribution of environmentally friendly manufacturing practices (Saad et al., 2019). The last hypothesis innovative culture positively moderates the impact on waste, energy, and resource management factors such as process and equipment, product design, corporate social responsibility, human resource practices, and manufacturing planning and control on sustainable performance. The findings reveal that innovative culture influences the linkages between energy, waste, and resource management and long-term sustainability. These findings suggest that having a stronger inventive culture in the company will increase the effects of process and equipment practices, as well as firm's relationships, on sustainable performance. As a result, it is critical for manufacturing company executives to conduct the necessary work to raise employee understanding of energy, waste, and resource management to create a welcoming environment for all workers.

## Conclusion

This study analyzed the effect of efficient resource management on sustainable performance and the moderating role of innovative culture in oil and gas firms in China. The study revealed that processes and equipment, human resource practice, manufacturing planning and control, product design, and corporate social responsibility have a significant impact on sustainable performance in oil and gas firms in China. Furthermore, innovative culture partially moderates the relationship between management practices and sustainable performance. The findings of the study have a number of implications for manufacturing executives as well as practitioners. Considering the sustainability impacts that inspire manufacturing firms to employ resource management practices would assist practitioners in adjusting plans and practices to management practices among industries and, as a result, reduce energy and resource consumption and wastage. The research is a solid basis for making policies and building innovative culture in manufacturing industries, from both a theoretical and practical point of view.

### Implications of Study

The purpose of this research was to produce theoretical and practical advancements in the realm of knowledge. Furthermore, this research provides policymakers, practitioners, and managers of manufacturing firms with relevant information in a variety of ways. The findings of the study have a number of implications for manufacturing executives as well as policymakers that modify this study to incorporate the waste, energy, and resource management practices that contribute to the firm's sustainable performance. The large positive benefits of waste, energy, and resource management relationships on company performance suggest that these resources should be linked to improve the firm's current sustainable performance. Managers should also focus on creating an innovative culture to improve the impact of human resource practices and corporate social responsibility on a firm's sustainable performance. From a research point of view, this study adds to “what is known about oil and gas manufacturing firms by showing how important it is to manage waste, energy, and resources to improve sustainable performance.” The study also contributes to the body of knowledge by looking at the impact of interactions between creative culture and waste, energy, and resource management on sustainable performance. As a result, managers and policymakers should develop a fair policy for implementing the understanding of innovative culture that motivates manufacturing firms to implement management practices. This will support practitioners in adjusting policies and initiatives to encourage waste, energy, and resource practices among businesses. Finally, the study established its own institutional theory into a particular framework. From an academic point of view, this research contributes to the current knowledge on resource manufacturing by identifying the importance of resource manufacturing techniques in improving a firm's long-term success. The research also adds to the body of knowledge by examining the effects of interactions between innovative culture and resource production techniques on long-term sustainability.

### Limitations and Future Study Direction

Even though the study achieves its goals, there are some limitations that must be noted before extrapolating its results. This study was conducted among employees of oil and gas firms in China. So, generalizing study results to other sectors may be an issue. The study sample is limited to China, and the data were gathered from several oil and gas manufacturing companies. Future research can evaluate the study's conceptual framework in other countries, allowing the findings to be implemented more globally. Furthermore, future research should focus on a specific industry, as the relationship between waste energy, and resource management and sustainable performance may differ depending on the industry in which manufacturing businesses operate. Even though researchers may use a longitudinal study design in the future to figure out cause and effect more accurately, the data were collected using a cross-sectional method. As a result, longitudinal research is required to get a better understanding of how management practices impact on sustainable performance. More research is needed to test the idea in other nations, both advanced and developing.

## Data Availability Statement

The original contributions presented in the study are included in the article/supplementary material, further inquiries can be directed to the corresponding author/s.

## Ethics Statement

The studies involving human participants were reviewed and approved by Jilin University, China. The patients/participants provided their written informed consent to participate in this study. The study was conducted in accordance with the Declaration of Helsinki.

## Author Contributions

SZ and SX collected the data. YW conceived, designed the concept, and wrote the paper. The authors read and agreed to the published version of the manuscript.

## Conflict of Interest

The authors declare that the research was conducted in the absence of any commercial or financial relationships that could be construed as a potential conflict of interest.

## Publisher's Note

All claims expressed in this article are solely those of the authors and do not necessarily represent those of their affiliated organizations, or those of the publisher, the editors and the reviewers. Any product that may be evaluated in this article, or claim that may be made by its manufacturer, is not guaranteed or endorsed by the publisher.
